# Current Trends in Vascular Biomarkers for Systemic Sclerosis: A Narrative Review

**DOI:** 10.3390/ijms24044097

**Published:** 2023-02-17

**Authors:** Bianca Saveria Fioretto, Irene Rosa, Marco Matucci-Cerinic, Eloisa Romano, Mirko Manetti

**Affiliations:** 1 Section of Anatomy and Histology, Department of Experimental and Clinical Medicine, University of Florence, 50134 Florence, Italy; 2 Section of Internal Medicine, Department of Experimental and Clinical Medicine, University of Florence, 50134 Florence, Italy; 3 Unit of Immunology, Rheumatology, Allergy and Rare Diseases (UnIRAR), IRCCS San Raffaele Hospital, 20132 Milan, Italy; 4 Imaging Platform, Department of Experimental and Clinical Medicine, University of Florence, 50134 Florence, Italy

**Keywords:** systemic sclerosis, scleroderma, vasculopathy, vascular damage, angiogenesis, biomarkers

## Abstract

Systemic sclerosis (SSc, scleroderma) is a multifaceted rare connective tissue disease whose pathogenesis is dominated by immune dysregulation, small vessel vasculopathy, impaired angiogenesis, and both cutaneous and visceral fibrosis. Microvascular impairment represents the initial event of the disease, preceding fibrosis by months or years and accounting for the main disabling and/or life-threatening clinical manifestations, including telangiectasias, pitting scars, periungual microvascular abnormalities (e.g., giant capillaries, hemorrhages, avascular areas, ramified/bushy capillaries) clinically detectable by nailfold videocapillaroscopy, ischemic digital ulcers, pulmonary arterial hypertension, and scleroderma renal crisis. Despite a variety of available treatment options, treatment of SSc-related vascular disease remains problematic, even considering SSc etherogenity and the quite narrow therapeutic window. In this context, plenty of studies have highlighted the great usefulness in clinical practice of vascular biomarkers allowing clinicians to assess the evolution of the pathological process affecting the vessels, as well as to predict the prognosis and the response to therapy. The current narrative review provides an up-to-date overview of the main candidate vascular biomarkers that have been proposed for SSc, focusing on their main reported associations with characteristic clinical vascular features of the disease.

## 1. Introduction

**Figure 1:**
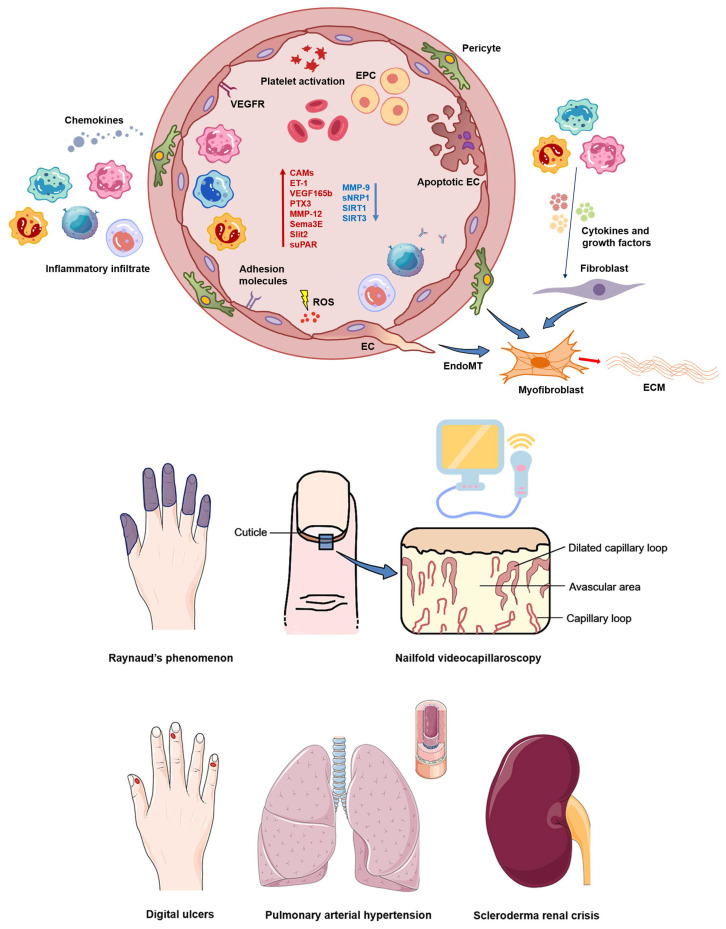
Main mechanisms underlying systemic sclerosis (SSc)-related vasculopathy. Vascular damage, representing a primary event in SSc pathogenesis, may be initiated by persistent activation or apoptosis of endothelial cells (ECs) as well as by multiple other factors that include autoantibodies, infectious agents, and reactive oxygen species (ROS). SSc-injured endothelium is characterized by increased expression of cell adhesion molecules (CAMs) and the production of several chemokines, all molecules that recruit immune cells and generate an overt perivascular infiltrate. Protracted inflammation finally results in an excessive activation of tissue-resident fibroblasts, which transdifferentiate into myofibroblasts and produce/release extracellular matrix (ECM) components, leading to tissue fibrosis. Notably, pro-fibrotic myofibroblasts may also originate from different cell types, including ECs via the so called endothelial-to-mesenchymal transition (EndoMT), and pericytes. Endothelial progenitor cell (EPC) dysfunction, persistent platelet activation, an imbalance between vasodilation and vasoconstriction, and altered circulating levels of both pro- and anti-angiogenic factors also participate in SSc vasculopathy. The earliest clinical manifestation of SSc-related microvascular impairment is represented by the dysregulation of the vascular tone control, evident as Raynaud’s phenomenon. Telangiectasias, pitting scars, periungual microvascular abnormalities (e.g., giant capillaries, hemorrhages, avascular areas, ramified/bushy capillaries) clinically detectable by nailfold videocapillaroscopy, ischemic digital ulcers, and pulmonary arterial hypertension generally occur later in the disease process. Chronic vasculopathy also plays a pivotal role in the most severe SSc-related renal vascular complication, i.e., scleroderma renal crisis. Hand, lung, kidney-2, and vascular-tunic-artery icons by Servier https://smart.servier.com/ (accessed on 12 December 2022) are licensed under CC-BY 3.0 Unported https://creativecommons.org/licenses/by/3.0/ (accessed on 12 December 2022).

Systemic sclerosis (SSc, scleroderma) is a multifaceted rare connective tissue disease, and its pathogenesis is dominated by immune dysregulation, small vessel vasculopathy, impaired angiogenesis, and both cutaneous and visceral fibrosis [1,2,3]. According to the peak extent of skin involvement, SSc can be clinically classified into two different groups, namely the limited cutaneous SSc (lcSSc) and diffuse cutaneous SSc (dcSSc) subsets, with the former characterized by a thickening of the skin that is limited to the face and areas distal to the elbows and knees, and the latter featuring cutaneous thickening even in the trunk and areas proximal to the elbows and knees. A growing body of evidence supports the concept that vascular damage represents a primary event in SSc pathogenesis, as it may precede skin and visceral fibrosis by months or years [4,5,6,7]. The earliest clinical manifestation of SSc-related microvasculopathy is represented by the dysregulation of vascular tone control, evident as Raynaud’s phenomenon (RP). Telangiectasias, pitting scars, periungual microvascular abnormalities (e.g., giant capillaries, hemorrhages, avascular areas, ramified/bushy capillaries) clinically detectable by nailfold videocapillaroscopy (NVC), ischemic digital ulcers (DUs), and pulmonary arterial hypertension (PAH) generally occur later in the disease process, severely affecting patients’ quality of life [1,2,3]. Chronic vasculopathy also plays a pivotal role in the most severe SSc-related renal vascular complication, i.e., scleroderma renal crisis (SRC), a clinical condition characterized by poor renal cortical perfusion and rapidly progressive renal failure [8,9]. Cardiac involvement, erectile dysfunction, vascular malformations of the gastrointestinal mucosa, and, to some extent, myopathy are also described as frequent [6]. In SSc, vascular injury may be initiated by persistent activation or apoptosis of endothelial cells (ECs) as well as by multiple factors, including autoantibodies, infectious agents, and reactive oxygen species (ROS) [1,2,3]. Once activated, ECs produce high levels of endothelin-1 (ET-1) and von Willebrand factor (vWF), and release low levels of nitric oxide and endothelial nitric oxide synthase, with a resulting imbalance between vasodilation and vasoconstriction that modifies the microvascular tone, thus contributing to tissue ischemia and chronic hypoxia [4,6,7,10]. ET-1 also triggers the activation of fibroblasts into myofibroblasts, thus promoting intimal hyperplasia, luminal narrowing, vessel obliteration, and consequent reduced capillary blood flow, while increased vWF levels stimulate platelet aggregation and hypercoagulation, leading to further vascular damage [4,6,7,10]. Notably, myofibroblasts may also originate from ECs via the so-called endothelial-to-mesenchymal transition (EndoMT) [11]. SSc-injured endothelium is also characterized by an increased expression of adhesion molecules and the production of several chemokines, all proteins that recruit immune cells and generate an overt perivascular infiltrate leading, over time, to tissue fibrosis [4,5,10]. The whole process is accompanied by insufficient compensatory mechanisms of angiogenesis, likely due to an imbalance between angiogenic (e.g., vascular endothelial growth factor (VEGF), matrix metalloproteinase (MMP)-9, endoglin, ET-1) and angiostatic (e.g., pentraxin 3 (PTX3), MMP-12, endostatin, angiostatin, semaphorin3E (Sema3E), and Slit2) factors, as well as by a defective vasculogenesis process caused by the reduction, dysfunction, and/or impaired recruitment of circulating bone marrow-derived endothelial progenitor cells (EPCs) [3,4,6,7,10]. A schematic representation of the main basic mechanisms underlying SSc-related vasculopathy is shown in Figure 1.

In SSc, plenty of studies highlighted the great usefulness in clinical practice of vascular biomarkers allowing clinicians to assess the evolution of the pathological process affecting the vessels, as well as to predict the prognosis and the response to therapy [10,12,13]. On these bases, the current review will provide an overview of the main candidate vascular biomarkers and their reported associations with SSc clinical vascular features.

## 2. Objective and Methods

In this article, we performed a comprehensive literature review on the main proposed vascular biomarkers for SSc on the bases of their distinct biological actions. In particular, we conducted a multiple database search (PubMed, Scopus, and Web of Science) for English language articles published between 1981 and 2023 using the following key words: “systemic sclerosis”, “scleroderma”, “vascular”, “serum”, “circulating”, and “biomarker”. From the references we found, we then subjectively selected a possible wide-ranging spectrum of suitable biomarkers. Some reference lists of identified articles were further articles that had been searched.

## 3. Cell Adhesion Molecules

Cell adhesion molecules (CAMs), cell surface proteins that help cells stick to each other and to their surroundings, can be divided into four major groups: (i) selectins, (ii) cadherins, (iii) members of the immunoglobulin superfamily, which are all associated with cell-cell adhesion, and (iv) integrins, which instead mediate cell-extracellular matrix (ECM) interactions. Cadherins and the immunoglobulin superfamily are homophilic CAMs, as they directly bind to the same type of CAMs on another cell, while integrins and selectins are heterophilic CAMs [14]. In the early stages of SSc, the increased expression of CAMs and the raise of their soluble forms have been reported to play important roles in the pathogenesis of vascular alterations, leading to EC activation, angiogenesis dysregulation, and chronic and progressive vascular damage [6,10,15]. On these bases, these molecules have been suggested as potential biomarkers of SSc-related vasculopathy.

### 3.1. Selectins

Selectins are a family of three structurally and functionally related molecules playing important roles in leukocyte cell homing and trafficking. L-selectin (also known as CD62L) is constitutively expressed on leukocytes, P-selectin is found on platelets and stored in Weibel-Palade bodies from where it is transported to the cell surface after EC activation, and E-selectin is expressed on ECs and megakaryocytes [16,17]. The best characterized ligand for the three selectins is P-selectin glycoprotein ligand-1 (PSGL-1), which is a mucin-type glycoprotein expressed on all white blood cells [16,17].

Contrasting results have been reported about circulating levels of soluble L-selectin in SSc patients, presumably due to the limited number of patients analyzed. In particular, some studies described decreased serum L-selectin in SSc with respect to healthy controls [18,19,20], while others found elevated soluble L-selectin values [21,22], with high levels positively associated with the presence of pitting scars and DUs [22].

Soluble E- and P-selectins have been proposed as useful biomarkers in various pathologic states in which platelets and/or ECs are activated [23,24]. In SSc, soluble E-selectin was associated with the presence of avascular areas detected with NVC in patients within the first 48 months of diagnosis, suggesting that it might be a useful biomarker of SSc activity [25]. In patients with early SSc, defined as subjects with RP together with SSc characteristic autoantibody profile and/or an NVC scleroderma pattern without any clinical manifestation of definite SSc, soluble E-selectin was higher in those who presented an NVC scleroderma pattern identified with megacapillaries and/or avascular areas [26].

### 3.2. Immunoglobulin-Like Cell Adhesion Molecules

Immunoglobulin-like CAMs belong to the immunoglobulin superfamily, one of the largest and most functionally versatile protein families comprising cell-surface and soluble proteins containing at least one immunoglobulin or immunoglobulin-like domain. The most well-known immunoglobulin-like CAMs include intercellular adhesion molecules (ICAMs), vascular cell adhesion molecules (VCAMs), and junctional adhesion molecules (JAMs), which are all important in leukocyte trafficking events [27]. When overexpressed, these adhesion molecules can be detected in a circulating soluble form, and can be considered markers of underlying endothelial activity and damage.

#### 3.2.1. Intercellular Adhesion Molecules and Vascular Cell Adhesion Molecules

ICAM-1 (CD54) and VCAM-1 (CD106), both induced by pro-inflammatory cytokines such as interleukin (IL)-1β and tumor necrosis factor (TNF)-α in response to endothelial damage, and both mediating firm leukocyte adhesion and transmigration across the vascular endothelium, have been intensively studied in SSc [12,28]. In particular, circulating levels of ICAM-1 were found to be significantly higher in SSc patients with DUs compared to those without [29], while elevated serum levels of VCAM-1 were not predictive for the occurrence of new DUs in SSc [30] and showed no association with NVC patterns [31]. Higher circulating levels of VCAM-1 were observed in SSc patients with PAH compared to both SSc without PAH and healthy controls [32]. A significant increase in VCAM-1 levels in SSc-PAH with respect to healthy subjects was also reported in a subsequent study, although no difference was detected between SSc patients with or without PAH, suggesting that this molecule cannot be considered as a biomarker of SSc-related PAH [33].

#### 3.2.2. Junctional Adhesion Molecules

JAMs are cell-cell adhesion molecules largely expressed on ECs, epithelial cells, fibroblasts, and circulating cells, which interact with other adhesion receptors on opposing cells through their extracellular domain, as well as with scaffolding and signaling proteins through their cytoplasmic domain [34,35]. Because of their multiple interactions, JAMs modulate cell adhesion, migration, epithelial and endothelial barrier formation, and angiogenesis, and they have been implicated in pathologic processes, including leukocyte recruitment to sites of inflammation, ischemia-reperfusion injury, and vascular permeability [34,35,36,37]. JAMs can also be detected in a soluble form in the circulation after the cleavage of the extracellular domain of the cell surface JAM [34,35,36,37]. Among these cell-to-cell adhesion molecules, JAM-A (also known as JAM-1/F11 receptor) and JAM-C (JAM-3), which have been both demonstrated to behave as pro-angiogenic molecules, represent the most investigated JAMs in SSc, with their extracellular domains being cleaved and released as soluble forms by a disintegrin and metalloproteinases (ADAMs) in inflammatory conditions [34,35,37,38]. In particular, circulating levels of both soluble JAM-A (sJAM-A) and soluble JAM-C (sJAM-C) were found to be significantly increased in SSc patients with respect to healthy controls, especially in those featuring early/active NVC patterns and ischemic DUs [36,37]. sJAM-C also revealed a good diagnostic accuracy in discriminating between SSc patients and controls and, compared to sJAM-A, demonstrated that it is a more suitable biomarker for more severe peripheral vasculopathy characterized by the development of ischemic DUs [36,37].

#### 3.2.3. Studies Combining Different Adhesion Molecules

Several studies have been performed to simultaneously correlate multiple soluble CAMs with SSc activity and progression. High levels of soluble adhesion molecules released by ECs, such as soluble E-selectin, ICAM-1, and VCAM-1, were found in the circulation of SSc patients with renal crisis, suggesting an activation of ECs in patients with this complication [39]. A significant reduction of the aforementioned adhesion molecules was reported in the sera of SSc patients after the infusion of iloprost, a prostacyclin analogue that is used for the treatment of SSc-related RP [40]. In another study, SSc patients with renal, myocardial and pulmonary involvement presented elevated serum E-selectin, VCAM-1, VEGF, and ET-1 when compared to both healthy controls and patients without systemic manifestations, indicating that circulating levels of these molecules may reflect the ongoing EC activation state and correlate to the extent of internal organ complications [41]. Finally, in a small cohort of SSc patients, increased serum soluble ICAM-1, VCAM-1 and P-selectin were demonstrated in SSc patients with PAH when compared to healthy controls [42]. Interestingly, circulating levels of all these molecules decreased to normal values after 12 months of therapy with bosentan, a dual ET-1 receptor antagonist [42].

## 4. Pro-Angiogenic Molecules

### 4.1. Vascular Endothelial Growth Factor

The VEGF family consists of numerous potent angiogenic regulators, including VEGF-A (and its different isoforms), VEGF-B, VEGF-C, VEGF-D, and placental growth factor [43,44]. In particular, VEGF-A (hereafter referred to as VEGF) represents one of the most potent regulators of the physiologic angiogenic process and has been found to be overexpressed in different pathologic conditions characterized by disturbed angiogenesis [43,44]. Several studies published during the last few years have demonstrated that, despite the lack of angiogenesis, VEGF levels are strongly elevated both in the skin and circulation of SSc patients [12,44,45,46,47,48,49,50]. In particular, since circulating VEGF was reported to be mainly increased in the early phases of the disease (defined as disease duration <5 years for lcSSc and <3 years for dcSSc), it was hypothesized that a prolonged VEGF upregulation, instead of promoting active angiogenesis, might represent a compensatory mechanism contributing to disturbed vessel morphology [10,49]. VEGF levels were also found to be significantly higher in SSc patients with systemic organ involvement [41] and to correlate with increased systolic pulmonary artery pressure (sPAP) [51,52], nailfold capillary density [51], and the presence of a late NVC pattern [31]. Moreover, SSc patients with DUs were found to show lower circulating VEGF levels than those without [49,53,54,55,56], suggesting that high VEGF could be protective against these ischemic complications when its concentrations exceed a certain threshold level [49]. Accordingly, raised VEGF serum levels did not predict the manifestation of new DUs [30], while low VEGF values were proposed as a risk factor for the occurrence of at least one new DU in SSc patients [54,55,56]. In other studies, both peripheral blood mononuclear cells (PBMCs) and activated platelets from SSc patients were found to produce and secrete significantly higher amounts of VEGF when compared to healthy controls [57,58]. In particular, it has been demonstrated that PBMCs from SSc patients produce increased quantities of VEGF in the early disease stages since, when compared to controls, only patients without active DUs and a less severe capillaroscopic pattern showed a significant difference in VEGF production [57]. Moreover, higher levels of VEGF in both platelet releases and plasma were reported in SSc patients with giant capillaries compared to those without [58]. Finally, in SSc patients, no correlations were found between levels of soluble VEGF receptor (VEGFR)-2 and DUs or nailfold bleeding [59].

### 4.2. Endoglin

Endoglin, also known as CD105, is a transmembrane glycoprotein highly expressed by activated ECs that plays a pivotal function in vascular development by acting as an accessory receptor for ligands of the transforming growth factor (TGF)-β superfamily. [60,61]. Although cell surface endoglin is pro-angiogenic, playing a role in vascular integrity and endothelium activity by mediating the vascular effects of TGF-β, the soluble form, represented by its extracellular domain cleaved by MMP-14, acts as an anti-angiogenic protein because it impairs the binding of TGF-β to its receptor, thus inhibiting endothelial nitric oxide synthase activation, and eventually deranging angiogenesis and promoting vasoconstriction [62,63]. When examining soluble endoglin levels in the serum of SSc patients, Fujimoto et al. found these levels to be higher in patients having lcSSc and telangiectasias [64]. Furthermore, in lcSSc patients, circulating endoglin values were positively correlated with sPAP, suggesting that an abnormal endoglin expression/function may be linked to lcSSc-specific manifestations [64]. Subsequently, raised endoglin serum levels were found in SSc patients compared to matched healthy controls, highlighting a possible contribution of this molecule in SSc vascular disturbances [62,65]. This hypothesis was supported by the results of a following multivariate analysis of a large SSc cohort showing an association between higher circulating endoglin and the SSc vascular phenotype characterized by the presence of DUs [62]. Similarly, an association between active DUs and increased endoglin was found in subsequent studies by Silva et al., although no predictive value for a new DU episode was reported [54,55,56]. Finally, when comparing serum endoglin according to the different NVC profiles, its circulating levels were found to be significantly higher in patients with a late NVC pattern compared to those with the early/active one [56].

### 4.3. Endothelin-1

ET-1, a potent endogenous vasoconstrictor peptide also mediating vascular wall cell proliferation, fibrosis, and inflammation, is mainly released by activated and/or damaged ECs through a mechanism triggered by protein kinase C activation [66]. In pathological conditions, ET-1 can be secreted by other several cell types, including fibroblasts, epithelial cells, smooth muscle cells, and inflammatory cells [66]. ET-1 exerts its biological activities by interacting with two different cell membrane receptors called ET receptor A (ETAR) and B (ETBR). The former is expressed by vascular smooth muscle cells and mediates vasoconstriction, while the latter is mainly expressed on ECs and mediates vasodilation [66,67]. Increased ET-1 levels have been demonstrated in the circulation of SSc patients [41,66,68], particularly in those with pitting scars and DUs [69,70,71]. However, in another pilot study, ET-1 values were demonstrated to be higher in SSc patients without DUs with respect to those with, with no association with development of new DUs [67]. Significant correlations were observed between ET-1 and single NVC measures, such as capillary number/dimension and ramified/enlarged capillaries, suggesting that this protein can be involved in the progression of SSc microvasculopathy [70]. ET-1 was also found to be significantly lower in the plasma of patients with the early NVC pattern when compared to those with the late one [72], while in a more recent study, even if the discovery cohort serum ET-1 was found to be higher in patients with the active NVC pattern with respect to those with the early and late ones, such a result was not confirmed in the replication cohort [31]. Increased ET-1 has been demonstrated in the circulation of SSc patients with PAH when compared to both healthy controls and SSc patients without PAH [68,73]. Interestingly, treatment with bosentan significantly decreased ET-1 in SSc patients with PAH to levels comparable to those in patients without, indicating that the levels of this peptide could reflect PAH presence as well as the severity and the response to bosentan therapy [73]. Serum ET-1 was also found to correlate with the echocardiographic parameters of right ventricular overload, which is considered a noninvasive indicator of right ventricular dysfunction in SSc patients [74]; while in SSc patients with SRC, several studies showed raised levels of ET-1 and increased expression of ETAR and ETBR [75,76,77]. SSc patients with internal organ involvement presented significantly higher levels of ET-1 compared to those without any evidence of systemic manifestations [41]. However, any relevant correlation between ET-1 serum measurement and the severity of organ involvement was found in a subsequent study [78]. Finally, the presence of elevated autoantibodies against ETAR, which is known to be associated with characteristic SSc features such as vascular, inflammatory, and fibrotic complications, was found to correlate with an increased risk of the development of PAH and DUs [79,80,81].

## 5. Anti-Angiogenic Molecules

### 5.1. VEGF165b

As already mentioned above, despite the lack of angiogenesis, pro-angiogenic VEGF levels are strongly elevated in SSc patients [12,31,41,44,45,46,47,48,49,50,51,52,53,54,55,56,57,58,59]. One possible explanation of such a paradox, according to which VEGF levels, rather than being associated with evidence of angiogenesis, actually correlate with progressive microvascular loss, seems to reside on the presence of different VEGF isoforms and to a switch from pro-angiogenic to anti-angiogenic variants. Indeed, it has been demonstrated that through an alternative splicing in its terminal exon, the VEGF primary transcript can produce at least six isoforms, one of which is represented by the anti-angiogenic VEGF165b splice variant [82,83,84], and that in SSc patients the increase in VEGF is the result of a significant rise in the anti-angiogenic VEGF165b isoform instead of the corresponding pro-angiogenic VEGF165 [85,86]. In particular, augmented VEGF165b circulating levels were shown to be both early and persistent features of the disease, and microvascular ECs isolated from SSc skin constitutively expressed and released higher levels of VEGF165b than those from healthy individuals [85]. It is worth noting that the plasma levels of VEGF165b were found to be significantly raised in SSc patients with the late NVC pattern compared to those with the early and active ones, and that they were increased in patients with either the active or late NVC pattern with respect to controls [87]. Moreover, augmented VEGF165b was reported to significantly correlate with the absence of microhaemorrhages and the presence of ramified/bushy capillaries and avascular areas [87].

### 5.2. Pentraxin 3

PTX3 is a soluble pattern recognition receptor belonging to the pentraxin superfamily and locally produced at inflammation sites by macrophages, dendritic cells, activated ECs, smooth muscle cells, and fibroblasts [88]. A consistent body of evidence has shown that PTX3 plays an important role in antimicrobial innate immunity, inflammation, ECM deposition, and neovascularization. Specifically, it binds to apoptotic cells and selected pathogens, it activates and modulates the classical complement pathway, and, by being a component of the ECM, it also contributes to fibrosis [88]. Finally, PTX3 acts as an anti-angiogenic factor by binding to fibroblast growth factor-2 (FGF-2) with high affinity and specificity, and suppressing FGF-2-dependent EC proliferation and neovascularization [88]. On the bases of its pleiotropic effects on inflammation and fibrosis as well as its inhibitory effect on neovascularization, PTX3 has been proposed as an intriguing candidate mediator in the pathogenesis of SSc. Circulating PTX3 levels were found to be elevated in SSc patients compared to controls [89,90,91]. Conversely, in a clinical observational study, PTX3 serum concentrations only tended to be elevated in SSc patients compared to healthy controls, as the difference between the two groups was not statistically significant [92]. Similarly, although circulating PTX3 was found to be lower in dcSSc patients compared to those with lcSSc, Ilgen et al. reported no difference in serum PTX3 between SSc and healthy cases [93]. Moreover, in the same study, PTX3 levels showed no correlation with the presence of DUs, RP, capillaroscopy findings, or sPAP measurements in SSc patients [93]. In contrast with such a result, two studies performed on SSc patients highlighted correlations between PTX3 and the presence or future development of vascular manifestations, including pitting scars, DUs, and PAH [89,90]. As a matter of fact, PTX3 was found to be elevated in SSc patients with DUs or PAH, and a multivariate analysis identified elevated levels of this protein not only as an independent parameter associated with the presence of DUs and PAH but also as a useful predictor of future occurrences of DUs [90]. It is worth noting that it was revealed that high concentration of PTX3 may inhibit EPC-mediated vasculogenesis [90]. Finally, with regards to vascular alteration, the authors observed in another in vitro study that PTX3 circulating levels were significantly higher in sera from newly diagnosed SSc patients than in those from healthy donors and cyclophosphamide-treated SSc patients [91].

### 5.3. Endostatin

Endostatin, represented by the cleaved carboxyl-terminal fragment of type XVIII collagen, is a potent angiostatic peptide that exerts its function by blocking VEGF activity. Indeed, it may be considered as an endogenous VEGF antagonist [94]. Endostatin has also been shown to be contained in platelet alpha granules, and to be released during platelet activation and aggregation [94]. Several studies investigated endostatin concentrations in SSc patients, and contradictory results have been reported [48,49,53,91,95,96,97,98,99]. Indeed, although most of them found significantly increased endostatin levels in SSc patients compared to healthy controls [48,53,91,95,96,97,98,99], one did not achieve such a result, reporting no substantial difference between SSc and controls [49]. In the disease course, several studies found that elevated endostatin positively correlated with ischemic manifestations, including pitting scars, DUs, and gangrene [53,95,100,101], while another failed to show any connection or predictive value of endostatin and DUs [55]. Similarly, as far as NVC is concerned, if numerous authors highlighted that endostatin serum levels increased with the progression of capillaroscopic damage [100,101,102], others couldn’t find any difference among the three NVC patterns [49,56,99]. Interestingly, higher levels of endostatin were reported in patients with avascular areas, while an inverse association between endostatin and giant capillaries, as well as microhaemorrhages, was demonstrated [49]. A positive relationship with endostatin circulating levels has been shown also for PAH [48,98], SRC [98,100], and heart involvement, represented by tachycardia, rhythm/conduction disturbances, ischemia, and right ventricular systolic pressure [48,96]. The evaluation of endostatin serum levels could thus represent a noninvasive, helpful examination of heart involvement in the course of the disease [96]. Of note, skin perfusion of hand showed a negative correlation with the serum level of endostatin, while a positive correlation existed between endostatin and Doppler indices of digital arteries [102]. Finally, morphological changes of digital arteries (narrowing and obstruction) have been shown to be characterized by an increase in serum endostatin levels, suggesting that this molecule could be a marker of skin perfusion and digital arteries damage of the hands [102]. Of note, again, a very recent proteomic aptamer analysis found endostatin as a circulating biomarker associated with disease progression from the very early/preclinical disease phase to definite SSc [103].

### 5.4. Angiostatin

Angiostatin is a proteolytic product of plasminogen with anti-angiogenic properties, as it antagonizes the trophic effects of several growth factors, including VEGF [104]. In ECs, it inhibits cell proliferation, migration, and capillary-like tube formation, and it induces the production of other anti-angiogenic factors, such as thrombospondin-1 [104]. As far as SSc is concerned, different studies reported higher serum levels of angiostatin in SSc patients with respect to healthy controls [91,99,105,106]. In particular, elevated angiostatin was observed in patients with more advanced stages of disease and with active and late NVC changes, allowing the authors to hypothesize that this molecule might be considered as a marker of late disease [106].

## 6. Angiopoietins

Angiopoietin (Ang)-1 and Ang-2 are two endothelial specific growth factors that have been demonstrated to interact with VEGF, contributing to the fate of blood vessels during angiogenesis [107]. The interaction of Ang-1/Ang-2 with the specific tyrosine kinase receptor Tie2 is believed to exert dual effects on vascular ECs, and regulate angiogenesis in a context-dependent manner. Indeed, the Ang/Tie2 system seems to be essential in controlling EC activation, sprouting angiogenesis, and vascular remodeling [107]. In a recent study, Michalska–Jakubus and colleagues reported that serum concentrations of Ang-1 were significantly decreased, while those of Ang-2 were increased, in SSc patients with respect to healthy individuals [107]. As far as peripheral vasculopathy is concerned, giant capillaries and microvascular leakage/collapse were associated with Ang-1 deficiency and concomitant increase in VEGF levels [107]. Moreover, SSc patients with the late NVC pattern presented elevated serum levels of both Ang-2 and VEGF [107]. On the basis of these intriguing findings, the authors proposed that Ang-1 deficiency may be involved in early capillary enlargement and subsequent collapse, followed by the formation of unstable new capillaries in a VEGF-enriched microenvironment, while circulating Ang-2 seems to increase later with SSc-related microvascular disease progression [107]. In this context, since the bi-specific antibody faricimab, which is simultaneously directed against Ang-2 and VEGF, has recently shown promising results in clinical trials for the treatment of patients with diabetic retinopathy [108], we believe it would be worth investigating its possible efficacy also in SSc.

## 7. Matrix Metalloproteinases and Tissue Inhibitors of Matrix Metalloproteinases

Connective tissue turnover strictly depends on the balance between ECM synthesis and degradation. ECM breakdown is mainly regulated by MMPs (MMP-1 to MMP-28), whose activity is in turn controlled by a family of homologous proteins, the tissue inhibitors of MMPs (TIMP-1 to TIMP-4) [109,110]. MMPs are essential for various physiological processes such as embryonic development, morphogenesis, angiogenesis, and cell migration, and they have been implicated in a number of key pathologic processes, including inflammation, fibrosis, arthritis, pulmonary diseases, and cancer [109,110]. The analysis of specific MMPs and TIMPs in the circulation of SSc patients suggests possible effects on outcomes in different tissues [109,110]. In a study investigating serum concentrations of MMP-9 in SSc patients with and without PAH and in those with PAH treated or not treated with bosentan, MMP-9 levels were reported to be significantly lower in patients with PAH, and to be up-regulated following bosentan treatment [111]. A significant decrease in circulating MMP-9 levels was also found in SSc suffering from ischemic retinopathy, thus suggesting MMP-9 as a novel predictive marker of such a microvascular complication [112]. Serum levels of MMP-12 were found to be significantly increased in SSc patients compared with controls [91,113], and to be associated with the presence of DUs and the severity of nailfold capillary abnormalities [113]. In a cross-sectional study, circulating TIMP-4 was reported to be significantly augmented in SSc patients with respect to healthy subjects [114]. In particular, in patients with sPAP measurements lower than 40 mmHg, TIMP-4 levels were comparable to those of controls, while individual sPAP measurements suggestive of PAH were found to be associated with increased TIMP-4 levels, indicating a cardiopulmonary vasculature-specific role of TIMP-4 activation in SSc [114]. Finally, a decrease in the concentration of MMP-3 and a parallel increase in both TIMP-1 and TIMP-2 were described in the plasma of SSc patients, although no associations with specific vascular manifestations were investigated [115].

## 8. Neurovascular Guidance Molecules

Neurovascular guidance molecules are proteins with attractive and repulsive properties that have been reported to regulate the sprouting of both nerves and blood vessels, and to be involved in different pathologic conditions, including tumor growth/metastasis and autoimmune diseases [116,117]. In SSc, members of semaphorin/plexin/neuropilin and slit/roundabout families have been recently associated with an impaired control of vascular tone, peripheral microvasculopathy, and defective angiogenesis, with their serum levels significantly correlated with different vascular manifestations [116,117,118,119,120,121,122].

Semaphorins, consisting in a large family of transmembrane and secreted proteins grouped into eight classes on the bases of their structural domains, can play a repulsive or attractive role depending on the cell types and the different biological context [116]. Cell-associated semaphorins bind to plexins, whereas secreted class III semaphorins (Sema3s) bind to neuropilins (NRPs), which function as plexin coreceptors and do not signal themselves. Sema3E represents the only exception, as it directly binds to PlexinD1 [116]. Sema3s have been shown to be able to regulate angiogenesis [116,123]. In particular, Sema3C has a bifunctional activity, being both a pro-angiogenic and an anti-angiogenic factor, while Sema3A, Sema3B, Sema3D, Sema3E, and Sema3F all exert anti-angiogenic properties [116,123]. In a study from our group, serum Sema3E levels were found not only to be increased in SSc patients compared to healthy individuals, but also to positively correlate with the early NVC pattern and the absence of DUs, suggesting that Sema3E might represent a biomarker of early vascular involvement [121].

NRPs (NRP1 and NRP2) are single-pass transmembrane, non-tyrosine kinase glycoprotein receptors that have an important role in several physiological processes and pathological conditions [116]. Since NRP2 is predominantly expressed by lymphatic ECs while NRP1 is present on the ECs of blood vessels [116], two studies performed by our group investigated the possible implication of NRP1 in SSc-related microvasculopathy [118,120]. In the former, serum levels of soluble NRP1 (sNRP1) were found to be significantly decreased in SSc patients with respect to healthy controls and to progressively decline within the SSc group, reaching the lowest values in those patients with the active and late NVC patterns [118]. In the latter, lower circulating sNRP1 levels were reported when compared to controls in both SSc patients and in the patients with a very early diagnosis of SSc (VEDOSS) [120,124]. Interestingly, sNRP1 levels were not statistically different between VEDOSS and SSc, suggesting that VEDOSS patients already show features typical of the established disease [120]. Finally, increased sNRP1 has been proposed as a potential biomarker to identify SSc patients at risk of developing PAH [119].

The slit family consists of three secreted glycoproteins (Slit1, Slit2, and Slit3) acting as ligands for transmembrane roundabout (Robo) receptors, namely Robo1, which is expressed in both the nervous and the vascular systems; Robo2 and Robo3, which are predominantly expressed in the nervous system; and Robo4, also called “magic roundabout”, which is a novel EC protein recently discovered [116]. Slit/Robo signaling has been implicated in both physiological and pathological angiogenesis. In particular, Slit2 has a bifunctional activity, as if it acts as a pro-angiogenic factor in a Robo1-dependent manner, with Robo1 being required for VEGF-induced phosphorylation of VEGFR-2 in ECs, and it may also exert anti-angiogenic effects by interacting with Robo4 [116]. The contribution of Slit2 to SSc-related vascular dysfunction has been demonstrated in a work published by our group, in which circulating Slit2 levels were found to be significantly increased in sera from both SSc and VEDOSS patients with respect to controls, with higher levels being specifically correlated with the presence of microvascular abnormalities in VEDOSS subjects, suggesting that Slit2 could reflect the presence of peripheral vascular impairment since the very early phase of SSc [122].

Although variations in the levels of sNRP1, Sema3E, and Slit2 have been proposed as a useful device to assess microcirculatory abnormalities at different stages of SSc, since all the aforementioned studies evaluated a single neurovascular guidance molecule at a time and in relatively small groups of patients, our group has performed additional research in order to simultaneously measure sNRP1, Sema3E, and Slit2 in a larger SSc cohort [117]. In agreement with previous findings, in this study, sNRP1 was reported to be significantly decreased in SSc, with lower levels correlating with the severity of NVC abnormalities and the presence of ischemic DUs [117]. Moreover, both Sema3E and Slit2 were found to be increased, with Sema3E better reflecting early NVC abnormalities and positively correlating with the absence of DUs, and with augmented Slit2 significantly associating with the occurrence of DUs [117]. Interestingly, the ROC curve analysis revealed that both sNRP1 and Sema3E serum levels have a moderate diagnostic accuracy, while the logistic regression analysis allowed us to distinguish these two molecules as better suited independent biomarkers reflecting the activity and severity of SSc-related peripheral microvasculopathy [117]. Moreover, the evidence that sNRP1 levels in SSc patients with an early NVC pattern are comparable to those in healthy controls but significantly higher than in patients with an active/late NVC pattern indicated that it might be employed as a marker of microvascular disease progression. Conversely, since Sema3E levels rise in patients with an early NVC pattern and then decrease with progression of microvascular abnormalities, it may be better suited as early diagnostic marker [117].

## 9. Sirtuins

Sirtuins (SIRTs) are a family of NAD-dependent protein deacetylases exerting pleiotropic effects on several biological processes, including metabolism, cell survival, and cell proliferation [125]. Among the seven SIRT isotypes (SIRT1–SIRT7), SIRT1, SIRT6, and SIRT7 are mainly nuclear, while others are mostly cytoplasmic (SIRT2) or mitochondrial (SIRT3, SIRT4, and SIRT5) [125]. Since SIRT1 and SIRT3 have recently emerged as noteworthy players in regulating angiogenesis [126,127,128], our group recently conducted a study in order to evaluate the association of these circulating deacetylases with the severity of SSc-related peripheral microvascular damage [129]. In this study, we provided the evidence that serum levels of both SIRT1 and SIRT3 are decreased in SSc patients compared to healthy controls and that their decrease correlated with the severity of NVC abnormalities, with SIRT3 also being related to the occurrence of ischemic DUs. Of note, via logistic regression analysis, we further demonstrated that, between the two SIRTs, SIRT3 may better mirror peripheral vascular disease activity and severity [129].

A summary of the aforementioned vascular biomarkers proposed in SSc is shown in Table 1.

## 10. Cytokines

Cytokines are a large category of peptides that include interleukins, chemokines, adipokines, interferons (IFNs), lymphokines, and TNFs. These small proteins are produced by a broad range of cells (immune cells like macrophages, B lymphocytes, T lymphocytes and mast cells, as well as ECs and fibroblasts), and given their inability to cross cell membrane to enter the cytoplasm, they act through cell surface receptors.

### 10.1. Interleukins

Interleukins can be grouped into four major clusters: (i) the IL1-like cytokines, consisting in seven members with agonistic functions (IL1α, IL1β, IL18, IL33, IL36α, IL36β, and IL36γ) and four members with antagonistic activities (IL1Ra, IL36Ra, IL37, and IL38); (ii) the class I helical cytokines (IL4-like, γ-chain, and IL6/IL12-like); (iii) the class II helical cytokines (IL10-like and IL28-like); and (iv) the IL17-like cytokines [130]. A fifth group comprises interleukins not fitting into any of the four others (e.g., IL35 and IL16) [130]. Given their pivotal role in the pathogenesis of SSc [131,132], several interleukins belonging to the IL1 family have been investigated as possible useful disease biomarkers. In contrast with the study by Hussein et al., in which a distinct elevation of serum IL1β was observed in SSc patients when compared to controls [133], in a more recent work no significant difference in serum concentrations of IL1β and IL1α was observed between SSc patients and healthy individuals, even if SSc subjects with high IL1α were more likely to have DUs [134]. Circulating IL18 levels were found to be significantly higher in SSc patients respect to controls [134,135] and, interestingly, serum levels of IL18-binding protein isoform a (IL18BPa), a soluble decoy receptor for IL18, were also found to be elevated in SSc circulation and to positively correlate with sPAP [136]. As far as IL33 is concerned, different studies reported a significant increase in its circulating levels in SSc [137,138,139,140,141], particularly in patients with DUs [137,141]. Notably, circulating values of soluble suppression of tumorigenicity 2 (ST2), a member of the IL1R/Toll-like receptor family that IL33 binds to, were also found to be higher in SSc patients and to correlate with diastolic dysfunction, sPAP, DUs, and NVC abnormalities [140,141]. In particular, ST2 was increased in SSc patients with diastolic dysfunction and lower in those with elevated sPAP [140]. Moreover, SSc patients showing DUs and a late NVC pattern had higher ST2 levels than those without DUs and presenting an active NVC pattern [141], with ST2 being associated with the development of new DUs [141]. Finally, SSc patients without proximal-distal gradient (PDG) at laser speckle contrast analysis had significantly higher ST2 compared to SSc patients with PDG [141].

Among class I and II helical cytokines, serum from SSc patients was reported to be characterized by considerably higher values of IL13, IL4, IL10 [142,143], IL6 (which positively correlated with both PAH and cardiac involvement) [144,145], IL22 [146], and IL35 [147,148], which was particularly increased in patients with an early NVC pattern [148]. In other studies, IL1β and IL13 have also been recorded to be significantly elevated in the serum and plasma of lcSSc patients with PAH [32,149], while IL6 levels, even if not significantly different between SSc and controls, were found to be lower in patients with DUs compared to those without [150]. When compared to healthy individuals, SSc patients were also found to present significantly elevated levels of the soluble form of the oncostatin M receptor (sOSMR), which has been thought to act as an antagonist of the IL-6 family-belonging oncostatin M [151]. Of note, since higher levels of sOSMR were present in patients with DUs compared to those without, the authors suggested sOSMR as a candidate biomarker of the disease [151].

Concerning IL17-like cytokines, no difference between SSc patients and healthy subjects has been reported with regards to the serum concentration of IL17A, while IL17B, IL17E, and IL17F were demonstrated to be significantly higher in SSc, with circulating IL17B being more elevated in those patients with renal abnormalities compared to those without [152]. Subsequently, IL17F and IL17E were found to correlate with the prevalence of DUs, whereas IL17F was reported to be associated with elevated right ventricle systolic pressure values [153]. Finally, even though IL17 levels did not significantly differ between SSc and controls, higher IL17 was reported in the serum of patients with telangiectasias compared to those without [150].

When considering other interleukins, IL16 was reported to be significantly elevated in SSc compared with healthy subjects [154], while, among SSc patients, IL32 was found to be higher in those with PAH and to correlate with sPAP [155]. Finally, the macrophage migration inhibitory factor (MIF), a pleiotropic cytokine with pro-inflammatory properties, has also been reported to be raised in SSc patients with PAH [156]. In a very recent multi-center prospective study, a multiplex array analysis allowed to identify cytokine profiles useful to distinguish SSc patients who are either at high-risk for or have PAH from SSc patients who may be at lower risk for PAH and healthy controls [157].

### 10.2. Chemokines

Chemokines are a family of small secreted chemotactic cytokines classified into four subfamilies according to the position of cysteine residues next to the amino terminus of their amino acid sequence: (i) the XC chemokines, containing a single N-terminal cysteine; (ii) the CC chemokines, with two adjacent cysteines near their amino acid terminus; (iii) the CXC chemokines, presenting two cysteines separated by another amino acid; and (iv) the CX3C chemokines, which have two cysteines divided by three amino acids [158,159]. Chemokine signaling is class restricted, as CC-chemokines activate CC receptors while CXC chemokines trigger CXC receptors [158,159]. Chemokines are known to mediate leukocyte chemotaxis and migration through the endothelium into organ tissues, leading to interactions between leukocytes and fibroblasts, thus actively contributing to inflammation and accumulation of ECM [158,159]. In addition, they have emerged as angiogenesis mediators as they recruit pro-angiogenic immune cells and endothelial progenitors to the neo-vascular niche or directly regulate EC function through their receptors [158,159]. In particular, the CXC family of chemokines shows different angiogenic activity according to the presence/absence of the ELR motif (Glu-Leu-Arg). Indeed, the CXC chemokines containing ELR, such as IL-8 (CXCL8) and growth-related oncogenes α, β and γ (CXCL1-3), are strong inducers of angiogenesis, whereas the CXC chemokines lacking ELR, including CXCL4 and monokine induced by IFN-γ (MIG, CXCL9), are strong angiogenesis inhibitors trough the binding of the CXCR3 receptor [160]. The levels of chemokines and their receptors have been found to be augmented both in the circulation and within inflamed tissue of patients, with different rheumatic diseases, including systemic lupus erythematosus, rheumatoid arthritis, and SSc [66].

#### 10.2.1. CCL2

CCL2, also known as monocyte chemoattractant protein-1 (MCP-1), is produced by macrophages, fibroblasts, and ECs and it is involved in leukocyte trafficking and activation as well as in angiogenesis modulation [161,162]. Increased levels of CCL2 were found in SSc patients compared to healthy controls [163,164,165,166,167], but no correlation was shown with peripheral vascular manifestations, including pitting scars, DUs, gangrene [163], and the NVC patterns [164]. Circulating CCL2 was reported to decrease in SSc patients after treatment with prostaglandin E1, although no difference in this chemokine levels was found in patients with/without DUs, teleangectasias, or according to NVC patterns, either before or after therapy [165]. In another study, CCL2 serum levels were found to be significantly elevated both in the lcSSc and in the dcSSc subsets, but when considering lcSSc patients no association was detected with PAH, although a trend for CCL2 reduction was reported after treatment with bosentan or prostacyclin analogues [166].

#### 10.2.2. CCL3 and CCL5

CCL3, known as macrophage inflammatory protein-1α (MIP-1α), and CCL5, also named regulated on activation, normal T cell expressed and secreted (RANTES), are pro-inflammatory chemokines involved in leukocyte recruitment to the site of inflammation, and they have also been implicated in the regulation of angiogenesis and metastasis [168,169]. CCL3 and CCL5 were found to be higher in SSc when compared to healthy controls, and significantly decreased in patients after infusion with prostaglandin E1 [165]. However, circulating levels of these two chemokines resulted similarly in patients with/without DUs or telangiectasias, or in patients stratified according to the three NVC patterns both before and after therapy [165].

#### 10.2.3. CCL13

CCL13, or monocyte chemoattract protein-4 (MCP-4), is a molecule that induces chemotaxis in monocytes⁄macrophages, T lymphocytes, and eosinophils by binding cell surface chemokine receptors such as CCR2, CCR3, and CCR5 [170]. It also plays an important role in different cell functions, including migration, invasion, motility, and proliferation [170]. Two different studies measured circulating CCL13 levels in SSc patients [171,172]. The former reported an increase in serum CCL13 in SSc patients when compared to healthy controls, but no correlation was found with clinical signs of vasculopathy such as pitting scars, telangiectasias, or DUs [171]. In the latter, similar CCL13 levels were detected in SSc patients and controls, with CCL13 not correlating with different clinical parameters, including DUs [172].

#### 10.2.4. CCL20, CCL21 and CCL23

CCL20, also known as liver activation regulated chemokine (LARC) or macrophage inflammatory protein-3α (MIP-3α), is a pro-inflammatory chemokine that has been involved, together with its receptor CCR6, in cancer metastasis and various autoimmune diseases [173]. Through CCR6, this chemokine may increase VEGF expression in cancer cells, or promote angiogenesis in ECs [174]. In a recent cross-sectional study, SSc patients showed significantly higher CCL20 than healthy controls, with its levels being positively correlated with mean pulmonary artery pressure (mPAP), suggesting that circulating CCL20 may be involved in the development of pulmonary vascular involvement [175].

CCL21, additionally called secondary lymphoid tissue chemokine (SLC), together with its corresponding receptor CCR7, is implicated in both the organization of the thymic architecture and homing of various T cell populations and antigen-presenting dendritic cells to lymph nodes [174]. They have also been found to mediate angiogenesis in cancer and in autoimmune diseases such as rheumatoid arthritis [176,177]. A recent study revealed that CCL21 levels were increased in SSc patients when compared with healthy controls, and that such levels were elevated even before the diagnosis of PAH [178]. Moreover, CCL21 concentrations positively correlated with PAH development and the occurrence of PAH-related events, suggesting that this chemokine might be a promising marker for predicting the risk of SSc-related PAH and PAH progression [178]. CCL21 association with SSc-PAH and its predictive value of PAH development were recently confirmed by the same research group in two new independent cohorts [179].

CCL23, also known as macrophage inflammatory protein 3 (MIP-3), is secreted by different immune cell types, including eosinophils, neutrophils, and monocytes [180]. Through the interaction with its ligand CCR1, this chemokine has a chemotactic effect on monocytes/macrophages, dendritic cells, lymphocytes, and ECs, and it is also able to promote angiogenesis [180]. Serum levels of CCL23 were found to be higher in SSc patients with respect to healthy individuals, and to associate with disease activity and shorter disease duration [181]. Moreover, CCL23 was significantly raised in SSc patients with PAH than in those without, while no correlation was found with the presence of pitting scars, telangiectasias, or DUs [181].

#### 10.2.5. CXCL4

CXCL4, predominantly produced by megakaryocytes and released from platelet α-granules upon platelet activation, exerts potent anti-angiogenic effects by inhibiting EC proliferation and migration [182,183]. Serum levels of this chemokine were reported to be markedly elevated in SSc patients with respect to healthy controls [183,184,185,186], particularly in very early SSc [183,187]. CXCL4 levels were also found to be higher in SSc patients with DUs respect to those without [183]. Interestingly, patients with higher baseline level of CXCL4 showed an increased prevalence of newly onset DUs within 6 months [183]. SSc subjects with an early NVC pattern also presented higher CXCL4 values than those with the active or late patterns [183]. Finally, CXCL4 negatively correlated with the mean number of nailfold capillaries [183].

#### 10.2.6. CXCL5

CXCL5, produced upon IL-1 or TNF-α stimulation by a variety of cells, including epithelial cells, keratinocytes, ECs, fibroblasts, neutrophils, and monocytes, is a pro-angiogenic chemokine able to promote the chemotaxis of neutrophils possessing angiogenic properties [160,188]. It has been involved in different pathological conditions characterized by altered angiogenesis [160,188]. CXCL5 serum levels were found to be significantly lower in both lcSSc and dcSSc patients with respect to healthy subjects [188]. In the same study, when dcSSc subjects were classified into three groups, i.e., early stage (disease duration of <1 years), mid stage (1–6 years), and late stage (>6 years), circulating CXCL5 was found to be uniformly decreased in early stage dcSSc with respect to mid-stage dcSSc and healthy controls. Moreover, in non-early dcSSc (≥1 year), lower CXCL5 levels were associated with the occurrence of DUs, suggesting that this chemokine may be a marker of DU development in mid and late dcSSc stage [188].

#### 10.2.7. Other CXCL Chemokines

Among the CXC family of chemokines, CXCL8 was found not only to be higher in SSc patients with respect to controls, but also to parallel the severity of the disease subset, although no correlation was found with the NVC patterns [164]. Anti-angiogenic CXCL9 and CXCL10 were higher in SSc sera [189,190], but no correlation was found between CXCL10 and peripheral vascular manifestations, such as pitting scars, DUs, and gangrene [163]. Serum concentrations of CXCL11, reported to be raised in early SSc patients when compared with healthy individuals, strongly associated with disease activity [190], while pro-angiogenic CXCL16 was elevated in SSc, particularly in patients with PAH [189,190]. Recently, when measuring circulating levels of CXCL4, CXCL8, and GDF15, a small chemokine belonging to the TGF-β superfamily, in another cohort of SSc patients, only GDF15 was found to be higher in SSc and to associate with PAH and dilated capillary loops at capillaroscopy [191].

#### 10.2.8. CX3CL1

CX3CL1 (fractalkine) can be found in a membrane-bound form on the surface of ECs after stimulation with pro-inflammatory cytokines such as IL-1, TNF-α and IFN-γ, or in a soluble form after cleavage by metalloproteases [12,192]. Membrane fractalkine interacts with its unique receptor CX3CR1 to support integrin-independent leukocyte adhesion, while soluble fractalkine acts as a chemoattractant for monocytes/macrophages, natural killer cells, and T cells expressing CX3CR1 [12,192]. Fractalkine/CX3CR1 interaction has been implicated in the modulation of angiogenesis and vascular inflammation [193]. Soluble fractalkine levels were found to be significantly raised in SSc sera [194,195,196,197], to associate with pitting scars and DUs [194], and to decrease in SSc patients after prostaglandine E1 infusion [195].

### 10.3. Adipokines

Adipokines are proteins with metabolic properties released by either adipocytes or preadipocytes, adipose tissue-infiltrated immune cells, or other cell types within adipose tissue [198,199]. These molecules are known to participate in inflammatory processes, and may also show immunomodulating, pro- or anti-fibrotic, and angiogenic effects [198,199].

#### 10.3.1. Adiponectin

Adiponectin is an insulin-sensitizing, antiatherogenic, and anti-fibrotic hormone that may act as a double-edged sword in inflammatory states, as it plays an anti-inflammatory role in diseases such as obesity, type 2 diabetes, and atherosclerosis, while it acts as a pro-inflammatory agent for the development of chronic kidney disease, bowel disease, and rheumatoid arthritis [200]. As recently confirmed by two different meta-analysis studies, circulating adiponectin levels have been found to be decreased in SSc [197,201], particularly in dcSSc patients [202,203,204,205,206,207]. A single study reported correlations with signs of vasculopathy, as patients with reduced adiponectin concentrations showed a higher prevalence of pitting scars [203]. Finally, adiponectin was shown to significantly increase in SSc patients after treatment with epoprostenol, a prostaglandin analogue with powerful vasodilator activity [208].

#### 10.3.2. Leptin

Leptin is a multi-functional hormone involved in metabolism, immune regulation, and tissue remodeling [198]. Leptin may activate monocytes, dendritic cells, and macrophages stimulating the production of pro-inflammatory cytokines, and it is involved in different pathologies such as obesity, type 1 diabetes, and systemic lupus erythematosus [198]. Interestingly, leptin has also been found to promote angiogenesis [209]. Contrasting results have been reported over the years about the levels of leptin in the circulation of SSc patients, presumably due to the limited number of patients analyzed. In particular, some studies reported decreased leptin in dcSSc [205,210], while in others it was found to be increased, particularly in patients with PAH [143,211,212]. No significant differences in serum leptin were found in other studies [207,213]. Recent meta-analyses revealed that circulating leptin values were similar in patients with SSc and healthy controls [201,214].

#### 10.3.3. Resistin

Resistin, predominately secreted by immune cells after stimulation with TNFα, IL-6, and IL-12, exerts pro-inflammatory properties and has been involved in the pathologenesis of different inflammatory diseases [208]. Resistin is also able to induce an angiogenic response in ECs by enhancing ET-1 production [208]. In a preliminary study, circulating resistin levels were found to be similar between SSc and controls and, when patients were classified into two groups (i.e., patients with high or normal resistin levels on the bases of healthy control levels), no difference between these two groups was demonstrated for the frequency of cutaneous vascular manifestations, including pitting scars, telangiectasia, and nailfold bleeding [215]. A subsequent meta-analysis also found that resistin was not significantly different between SSc patients and healthy subjects [201]. Conversely, following studies demonstrated an increase in resistin values in SSc patients with respect to controls [143,197,208,216,217], with higher resistin in patients with DUs [213], in patients developing new DUs after a 52-weeks follow-up [217], and in those with PAH [143]. In another study, no association was found between resistin and nailfold capillaroscopy characteristics or DUs [216].

#### 10.3.4. Other Adipokines

Galectins, a family of proteins able to bind β-galactosides, play different biological functions in both physiological and pathological processes including angiogenesis, cell adhesion, inflammation, and immune cell regulation [218]. In SSc, higher concentrations of galectin 1 were associated with the presence of telangiectasias [219], while lower galectin 1 levels were present in patients with pitting scars/DUs, suggesting that this molecule may be a protective factor against the development of SSc-related digital vasculopathy [220]. Compared to control subjects, galectin 3 was also found to be higher in SSc patients [221]. Moreover, serum galectin-3 was reported to be significantly raised in patients with DUs [222].

Vaspin is an adipokine implicated in vascular inflammation and remodeling [223]. Although no difference in its circulating levels has been found between SSc and controls, vaspin was reported to be significantly decreased in SSc patients with DUs compared with those without DUs, while it did not correlate with SRC [223].

High circulating levels of adipsin, one of the major adipokines expressed by adipocytes, were found to significantly associate with PAH, and it was proposed as a novel adipose tissue-derived marker of SSc-related PAH [207].

Chemerin is an adipokine expressed in adipose tissue, dendritic cells, and macrophages that exerts a dual (“chimeric”) effect on inflammation, being able to be both pro- and anti-inflammatory, and behaving as a pro-angiogenic factor [224]. In the first study performed on 64 SSc patients, Akamata et al. found no difference in circulating chemerin levels between SSc and controls, but this adipokine was reported to be significantly higher in patients with DUs as compared with those without DUs [225]. Conversely, Sawicka et al. reported increased chemerin in SSc, but without any association with DUs or NVC patterns [224]. These results are in line with a previous study showing comparable chemerin levels when SSc patients were grouped into the three different NVC patterns or for the presence/absence of DUs [226]. Moreover, chemerin levels negatively correlated with PAH in dcSSc [224]. Interestingly, a recent proteome analysis on the circulation of several SSc subjects demonstrated that in SSc-PAH patients, chemerin was differentially expressed and significantly correlated with pulmonary vascular resistance (PVR) [227]. These results were validated by enzyme-linked immunosorbent assay in an independent cohort, leading the authors to conclude that chemerin might be an interesting surrogate biomarker for PVR in SSc-PAH [227].

Visfatin is a key regulator of metabolism and insulin resistance as well as an immunomodulatory and pro-inflammatory adipokine [224]. It also may attenuate angiogenesis and EC reparation [224]. No difference in visfatin concentrations was found between SSc patients and healthy subjects, and no association was reported with DUs or NVC patterns [224]. However, serum visfatin showed a positive correlation with PAH in patients with early disease [224].

### 10.4. Interferons

Serum IFN-γ levels were found to be higher in SSc when compared to controls and to be associated, among patients, with PAH [150]. Moreover, a higher type 1 IFN signature at both transcript and protein levels in whole blood or plasma of SSc patients has been found to correlate with more severe vascular manifestations [228].

A summary of the main cytokines proposed as potential vascular biomarkers in SSc is shown in Table 2.

## 11. Other Molecules Markers of Vascular Damage

### 11.1. Soluble Thrombomodulin

Thrombomodulin (TM), also known as CD141, is a type I transmembrane glycoprotein expressed on the surface of ECs that functions as a cofactor in the thrombin-catalyzed activation of protein C in the anticoagulant pathway by forming a 1:1 stoichiometric complex with thrombin [229]. Circulating fragments of TM lacking the transmembrane domain and known as soluble TM (sTM) are also found in blood, urine, and other biofluids [229]. In healthy humans, sTM levels are low, while high sTM levels are common in patients suffering from various diseases. In particular, a consistent increase in sTM levels is now widely regarded as an important biomarker for EC dysfunction and vascular risk assessment [229]. As far as SSc is concerned, sTM was found to be elevated in SSc patients respect to healthy controls [230,231], and to be significantly associated with PAH [232]. Differently, a previous study reported a significant decrease in circulating sTM in SSc patients with PAH compared to healthy controls [233].

### 11.2. Soluble CD163

CD163, the high affinity scavenger receptor for the hemoglobin-haptoglobin complex, is involved in the removal of necrotic cells, apoptotic cells, cell debris, and opsonized pathogens [234]. It is expressed on the cell surface of activated M2 macrophages, but it can be released from the membrane by proteolysis as a soluble form (sCD163) in response to oxidative stress or inflammatory stimuli [234]. In SSc patients, several studies have reported significantly elevated serum levels of this soluble molecule compared to healthy controls, suggesting that sCD163 might represent a useful potential biomarker for the disease [235,236,237]. In particular, patients with elevated serum sCD163 levels showed not only higher right ventricular systolic pressure and lower carbon monoxide diffusing capacity levels [235], but also a significantly higher sPAP [236], suggesting a possible association between sCD163 and the severity of disease-related PAH. Of note, elevated serum levels of sCD163 in SSc patients were reported to positively correlate also with renal vascular damage [238]. As far as DUs is concerned, conflicting results have been reported. Indeed, although increased sCD163 has been described to be indicative of a lower risk of DUs [239], in a previous report sCD163 serum levels were found to be significantly elevated in SSc patients with DUs compared to those without these complications [236]. Finally, another study demonstrated raised ex vivo production of sCD163 by PBMCs from SSc patients relative to healthy controls [240]. Although these findings collectively support sCD163 as a potential biomarker in SSc, it has to be considered that in a recent study such a soluble molecule did not emerge as a useful biomarker for specific SSc-related clinical manifestations [237].

### 11.3. Basigin

Basigin, also known as CD147 or extracellular MMP inducer (EMMPRIN), is a transmembrane glycoprotein belonging to the immunoglobulin superfamily that induces the synthesis of MMPs [241]. Serum soluble CD147 (sCD147) has been investigated to determine its possible role in SSc-related SRC pathogenesis. In particular, one study reported that sCD147 levels were significantly elevated in sera from SSc patients compared with healthy individuals, and that patients with elevated sCD147 had SRC more often than those with normal sCD147 levels [242].

### 11.4. Brain Natriuretic Peptide and N-terminal pro-Brain Natriuretic Peptide

Brain natriuretic peptide (BNP) belongs to the family of natriuretic peptides, that comprises also atrial, C-type, D-type, and V-type natriuretic peptides, as well as urodilatin. BNP, which is released into the blood from ventricular myocytes and fibroblasts in response to pressure overload and increased myocardial pressure, is synthesized as a longer precursor (proBNP) that is subsequently cleaved into an active C-terminal peptide (BNP), able to stimulate diuresis in order to reduce ventricular preload, and an inactive N-terminal fragment (NT-proBNP) [243]. Several studies have been conducted to evaluate the utility of NT-proBNP concentrations as a screening tool for SSc-related PAH [243,244,245,246,247,248,249,250,251,252,253]. A systematic review and meta-analysis through PubMed, Embase, and Cochrane Library databases was recently performed to estimate the diagnostic accuracy of serum NT-proBNP measurement in the clinical setting of SSc-PAH [254]. In this meta-analysis, involving many of the aforementioned studies [244,245,246,247,248,251,252,253], the pooled sensitivity and specificity of NT-proBNP to diagnose SSc-PAH were found to be 0.84 and 0.68, respectively, revealing that NT-proBNP has certain diagnostic value for PAH due to its better specificity and moderate sensitivity. However, although elevated serum NT-proBNP levels could warn clinicians of the occurrence of PAH, echocardiography and right heart catheterization should be conducted appropriately for further diagnosis [254].

### 11.5. Von Willebrand Factor

vWF is a circulating glycoprotein syntetized by injured ECs that plays an important role in the coagulation cascade by acting as a carrier and stabilizer for coagulation factor VIII. SSc patients have been found to exhibit increased vWF circulating levels with respect to healthy controls [255,256,257,258,259]. Moreover, it has been reported that raised serum vWF concentrations in lcSSc patients increase the risk of developing subsequent PAH [32,260]. However, a previous study did not find any difference between SSc patients with or without PAH [42].

### 11.6. Maresin 1

Macrophage mediators in resolving inflammation (Maresins) belong to the superfamily of specialized pro-resolving mediators, namely anti-inflammatory endogenous mediators derived from essential polyunsaturated fatty acids precursors, and are specifically synthesized by macrophages from docosahexaenoic acid [261]. Maresin 1 is principally produced by M2 macrophages, and it promotes a phenotype switch from pro-inflammatory M1 to M2 macrophage, with the consequent production of anti-inflammatory cytokines and growth factors [262]. In a very recent study, maresin 1 levels were found to be significantly lower in SSc patients respect to healthy controls [262]. In addition, during a follow-up period of 18 weeks, maresin 1 was reported to be higher in SSc patients with new DUs than in those without, suggesting a predictive role of this molecule in the development of these peripheral vascular manifestations [262].

### 11.7. Soluble Urokinase Plasminogen Activator Receptor

The urokinase plasminogen activator receptor (uPAR) is a key component of the fibrinolytic system, and plays an important role in ECM remodeling and angiogenesis. An abnormal uPAR cleavage resulting in dysfunctional uPAR-mediated pathways in ECs is implicated in SSc-related microvascular abnormalities and impaired angiogenesis [263]. Systemic levels of soluble uPAR (suPAR), which is released from cell membrane-bound uPAR, have been shown to positively correlate with both prognosis and mortality in a wide range of diseases [264]. As far as SSc is concerned, two studies have assessed circulating suPAR as a potential indicator of microvascular manifestations in SSc [265,266]. In the former, suPAR values were found to be higher in SSc patients than in controls, and to correlate with the presence of microvascular lesions including naifold capillaroscopic abnormalities and DUs [265], while in the latter suPAR levels were not associated with any vascular manifestation [266].

## 12. Concluding Remarks

Microvascular impairment is a key feature of SSc and represents the initial event of the disease, accounting for the main disabling and/or life-threatening clinical manifestations, including ischemic DUs, nailfold capillary abnormalities, PAH and SRC. Despite the various available therapeutic options, treatment of SSc-related vascular disease remains difficult, even considering SSc etherogenity and the quite narrow therapeutic window. In this context, the identification of reliable predictive biomarkers of vascular severity or extension becomes extremely relevant for the stratification of patients according to the risk and to allow an earlier therapeutic intervention, as well as for the development of novel specific therapies. Although several circulating SSc biomarkers of microvascular damage have been proposed so far, none of them have yet been validated and incorporated into treatment guidelines. Indeed, it is important to consider that: (i) blood-based biomarkers are not always perfectly specific for a given cell type or tissue; (ii) since sample collection/processing, as well as assay reagents, standards, and instrumentation for most biomarkers are not rigorously standardized, it is not correct to interpret biomarker values provided across studies with unidentical measurement techniques as “absolute”; and (iii) the lack of longitudinal studies for some biomarkers reduces the clinical impact of the findings. Interestingly, a recent narrative review highlighted that specific SSc-related autoantibodies and NVC patterns, so far considered independent prognostic markers for patients, appear instead to be interconnected, with a faster microvascular progression and certain autoantibody profiles characterizing patients with worse clinical outcomes [267]. In this context, the assessment of SSc autoantibodies and NVC profiles in combination with some of the most relevant herein discussed circulating vascular biomarkers could represent an additional tool to improve the accuracy of early diagnosis and guide targeted therapy. Of note, too, is that since multiplexed assays with a machine learning approach have been recently used to explore the association between different candidate vascular biomarkers and both DUs and PAH [119,268,269], it is likely that new biomarkers will be discovered and validated, thus making precision medicine in SSc a reality. Finally, large, multicenter, prospective studies of well-defined clinical cohorts should be performed to fully establish useful and reliable biomarkers of SSc-related vasculopathy.

## Figures and Tables

**Table 1:** Summary of the main vascular biomarkers proposed in SSc.

Molecule	Proposed Biomarker	Vascular Association	References
Cell Adhesion Molecules	↑ L-Selectin	pitting scars, DUs	[22]
	↑ E-Selectin	avascular areas at NVC megacapillaries/avascular areas at NVC SRC	[25] [26] [39]
	↑ P-Selectin	PAH	[42]
	↑ ICAM-1	DUs SRC PAH	[29] [39] [42]
	↑ VCAM-1	PAH SRC	[32,42] [39]
	↑ sJAM-A and sJAM-C	early/active NVC, DUs	[36,37]
Pro-Angiogenic Molecules	↑ VEGF	sPAP nailfold capillary density late NVC pattern	[51,52] [51] [31]
	↓ VEGF	DUs	[49,53,54,55,56]
	↑ Endoglin	telangiectasias, sPAP DUs late NVC pattern	[64] [62] [56]
	↑ Endothelin-1	pitting scars and DUs absence of DUs active NVC pattern PAH SRC	[69,70,71] [67] [31] [68,73] [75]
	↓ Endothelin-1	early NVC pattern	[72]
Anti-Angiogenic Molecules	↑ VEGF165b	late NVC pattern, absence of microhaemorrhages, presence of ramified/bushy capillaries and avascular areas	[87]
	↑ Pentraxin 3	pitting scars, DUs and PAH	[89,90]
	↑ Endostatin	pitting scars, DUs, gangrene progession of capillaroscopic damage avascular areas PAH SCR	[53,95,100,101] [100,101,102] [49] [48,98] [98,100]
	↓ Endostatin	giant capillaries, microhaemorrhages	[49]
	↑ Angiostatin	active and late NVC patterns	[106]
Angiopoietins	↓ Ang-1	giant capillaries, microvascular leakage/collapse	[107]
	↑ Ang-2	late NVC pattern	[107]
MMPs and TIMPs	↓ MMP-9	PAH ischemic retinopathy	[111] [112]
	↑ MMP-12	DUs, severity of nailfold capillary abnormalities	[113]
	↑ TIMP-4	sPAP	[114]
Neurovascular Guidance Molecules	↑ Sema3E	early NVC pattern, absence of DUs	[117,121]
	↓ sNRP1	NVC severity, DUs	[117,118]
	↑ sNRP1	PAH	[119]
	↑ Slit2	DUs	[117]
Sirtuins	↓ SIRT1 and SIRT3	NVC severity	[129]
	↓ SIRT3	DUs	[129]

DUs, digital ulcers; NVC, nailfold videocapillaroscopy; PAH, pulmonary arterial hypertension; sPAP, systolic pulmonary artery pressure; SRC, scleroderma renal crisis; SSc, systemic sclerosis.

**Table 2:** Principal cytokines proposed as potential vascular biomarkers in SSc.

Cytokines	Proposed Biomarker	Vascular Association	References
Interleukins	↑ IL1α	DUs	[134]
	↑ IL18BPa	sPAP	[136]
	↑ IL33	DUs	[137,141]
	↑ ST2	DUs, late NVC pattern	[141]
	↓ ST2	sPAP	[140]
	↑ IL13, IL4, IL10, and IL6	PAH	[144]
	↑ IL35	early NVC pattern	[148]
	↑ IL1β and IL13	PAH	[32,149]
	↓ IL6	DUs	[150]
	↑ sOSMR	DUs	[151]
	↑ IL17F and IL17E	DUs	[153]
	↑ IL17	telangiectasias	[150]
	↑ IL32	PAH and sPAP	[155]
	↑ MIF	PAH	[156]
Chemokines	↑ CCL20	mPAP	[175]
	↑ CCL21	PAH	[178,179]
	↑ CCL23	PAH	[181]
	↑ CXCL4	DUs, early NVC pattern	[183]
	↓ CXCL5	DUs	[188]
	↑ CXCL16	PAH	[189,190]
	↑ GDF15	PAH, dilated loops at NVC	[191]
	↑ CX3CL1	pitting scars, DUs	[194]
Adipokines	↓ Adiponectin	pitting scars	[203]
	↑ Leptin	PAH	[143,212]
	↑ Resistin	DUs PAH	[213,217] [143]
	↑ Galectin 1	telangiectasias	[219]
	↓ Galectin 1	pitting scars, DUs	[220]
	↑ Galectin 3	DUs	[222]
	↓ Vaspin	DUs	[223]
	↑ Adipsin	PAH	[207]
	↑ Chemerin	DUs	[225]
	↓ Chemerin	PAH	[224]
	↑ Visfatin	PAH	[224]
Interferons	↑ IFN-γ	PAH	[150]

DUs, digital ulcers; NVC, nailfold videocapillaroscopy; mPAP, mean pulmonary artery pressure; PAH, pulmonary arterial hypertension; sPAP, systolic pulmonary artery pressure; SRC, scleroderma renal crisis; SSc, systemic sclerosis.

## Data Availability

Not applicable.
